# Health-Related Quality of Life of Children and Adolescents With Congenital Hyperinsulinism – A Scoping Review

**DOI:** 10.3389/fendo.2021.784932

**Published:** 2021-12-03

**Authors:** Kaja Kristensen, Julia Quitmann, Stefanie Witt

**Affiliations:** Department of Medical Psychology, University Medical Center Hamburg-Eppendorf, Hamburg, Germany

**Keywords:** Health-related quality of life, congenital hyperinsulinism, children, adolescents, patient-reported outcomes

## Abstract

**Introduction:**

Despite improvements in diagnosis and therapeutic advances in treatment, congenital hyperinsulinism (CHI) remains a severe disease with high patient impairment. We aimed to review the literature on Health-related Quality of Life in children and adolescents with congenital hyperinsulinism and summarize the findings.

**Materials and Methods:**

For this scoping review, a literature search was conducted in PubMed and Web of Science in May 2021. Inclusion and exclusion criteria for the selection of articles were defined a priori.

**Results:**

Two hundred and forty-five (245) articles were identified through the search and screened on the basis of title and abstract. The full texts of forty articles were then assessed. Finally, four articles (published 2012-2020) describing Health-related Quality of Life in children and adolescents with congenital hyperinsulinism were included. The study designs were heterogeneous and included cross-sectional observational studies (n=2), clinical trials (n =1), and case reports (n=1) with different sample sizes. Three studies were conducted in European countries and one in Japan. The results for Health-related Quality of Life revealed inconsistencies.

**Conclusion:**

There are only a few studies looking at Health-related Quality of Life in children and adolescents with congenital hyperinsulinism. To gain a comprehensive understanding of the impact of congenital hyperinsulinism on Health-related Quality of Life in children and adolescents, it is necessary to use both generic and condition-specific instruments to measure Health-related Quality of Life of young patients in larger samples, to collect longitudinal data, and to consider qualitative research approaches.

## Introduction

Congenital hyperinsulinism (CHI) is a heterogeneous disorder that results in excessive, often unregulated, insulin secretion from pancreatic beta cells. Clinical manifestations range from life-threatening hypoglycaemia in newborns to mild symptomatic hypoglycaemia in early childhood, adolescence or adulthood, which can be difficult to identify (1).

Although CHI is a rare chronic health condition, it is the most common form of severe recurrent hypoglycaemia in infancy and childhood (1).

Due to the combination of hypoglycaemia and hypoketonaemia at an early age when neurons and neuronal networks are vulnerable to metabolic maladaptations, CHI is associated with abnormal neurodevelopmental outcomes with a tendency toward motor and language delays in early childhood (2). Incidence rates of abnormal neurodevelopmental outcomes varying between 26 and 44% have been reported for CHI patients (2, 3).

Also, the treatment can be challenging for patients. Careful glucose monitoring and daily oral or subcutaneous medication are strategies to manage CHI (4). However, oral diazoxide, the only drug for long-term treatment of hyperinsulinemic hypoglycaemia approved by the Food and Drug Administration (FDA), can lead to varying side effects such as excess hair growth, poor appetite, and fluid retention (5). Dietary management may also be required for reliable glucose delivery and the prevention of hypoglycaemia. However, feeding problems such as difficulty in swallowing, vomiting, and refusal to eat can complicate the management of hypoglycaemia, making tube feeding necessary (6).

When CHI is diagnosed, and normoglycaemia is achieved quickly, most children develop a normal range of cognitive, emotional, and social abilities. In these cases, complete clinical remission has been observed in medically responsive patients after several years of intensive conservative treatment (7).

A near-total pancreatectomy may be necessary for the most severe diffuse forms of CHI, in which abnormal beta cells are distributed throughout the pancreas and cannot be treated successfully with medication. However, as the size of the resection increases, the risk of surgery-related sequelae increases, particularly the possible development of pancreatic exocrine dysfunction, persistent hyperglycaemia or insulin-dependent diabetes. These complications of surgical therapy can also be observed with a latency of several years, e.g. with the onset of puberty (8).

Due to the above described consequences, both disease and treatment related, CHI may have a substantial impact on the Health-related Quality of Life (HrQoL) of those affected. The term HrQoL is a subjective, self-assessed and multidimensional construct that describes a person’s perception of his or her own physical, psychological, and social health status (9). This includes the impact of illness and treatment on the perception of health.

In the absence of a review on HrQoL in children and adolescents with CHI, this article aims to provide an overview of the current literature with the aim to improve our understanding of HrQoL in young CHI patients and to enhance the appropriate use of HrQoL instruments in research and practice.

## Methods

### Search Strategy and Eligibility Criteria

On May 19, 2021 we conducted a literature search in the databases PubMed and Web of Science which we updated it until September 23, 2021. The methodological framework of Arksey and O’Malley’s five-stage scoping review formed the basis for the literature search approach (10).

This review considers both qualitative and quantitative studies which focus on HrQoL in children and adolescents (aged 0 to 18 years) diagnosed with CHI. A prerequisite for inclusion in the review is that HrQoL was measured. The study design is therefore considered secondary as long as HrQoL was measured quantitatively or qualitatively in the studies. Thus, the review finds empirical studies such as cross-sectional studies, longitudinal studies, clinical trials, case reports or case series, and meta-analyses. No geographical, language, or time restrictions were placed. However, only studies that are published in peer-reviewed journals are included. These inclusion and exclusion criteria were defined a priori.

Since HrQoL is sometimes confused with or used as a synonym for other constructs, terms such as mental health, psychosocial health, and well-being were also considered. The complete search strategy included a combination of keywords and MeSH terms: (“Congenital Hyperinsulinism” [Mesh] OR “congenital hyperinsulinism”) AND (child* OR youth* OR teen* OR adolescent* OR infant* OR juvenile) AND (“quality of life” [Mesh] OR well-being OR psychosocial OR “mental health” OR “quality of life”).

### Selection Process and Data Extraction

After conducting the database search, exporting results, and removing duplicates, two independent reviewers screened titles and abstracts. The full texts of the articles that met the eligibility criteria or the articles for which it was not possible to assess eligibility based on the title and abstract were retrieved for further assessment. The same two independent reviewers evaluated all full-text articles against eligibility criteria. Any disagreements or uncertainties about eligibility were discussed until a consensus was reached.

A data extraction form was used for the final set of included studies: study population and setting; study design; the number of participants; participant characteristics; outcome measure; and the main results.

## Results

### Study Selection

**Figure 1:**
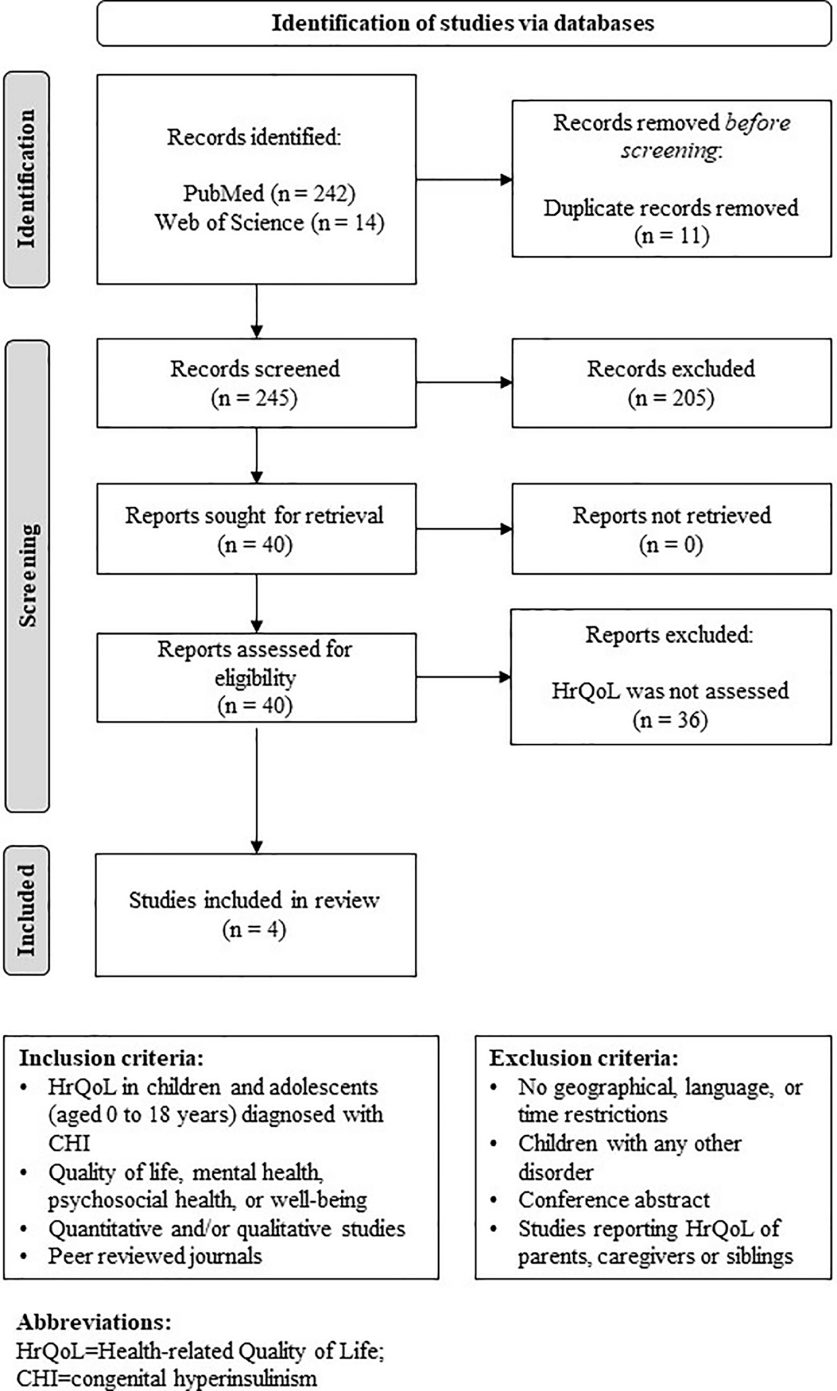
PRISMA flowchart of study selection and inclusion process.

The database search yielded a total of 245 citations. After title and abstract screening, 40 full texts were reviewed, of which four met the eligibility criteria and were included (11–14). The study selection process is shown in **Figure 1**, which is based on the PRISMA Statement (15).

### Study Characteristics

The four studies included in this scoping review were published between 2012 and 2020 (11, 14). They were mainly conducted in Europe (Finland, France, UK) (11–13) with the exception of Japan (14). Study designs include one cross sectional and one retrospective survey (12, 14), a longitudinal observational study (11), and a case report (13). The sample sizes ranged between one participant (13) and 447 participants (14). Instruments used to measure HrQoL of children and adolescents with CHI were the generic patient-reported outcome measures (PROMs) KINDL-R (12), PedsQL (13), AUQUEI picture questionnaire, and the QUALIN questionnaire (11) as well as a physician-reported questionnaire (14). A summary of study characteristics is provided in **Table 1**.

**Table 1:** Summary of included studies.

Author; Country	Aim	Study Design	Study Population	HrQoL Measures	Results
Le Quan Sang et al. (11); France	To describe changes in various outcomes after replacing three daily octreotide injections by a monthly injection of long-acting release octreotide in CHI patients	clinical trial, pre-post	n = 10; age: 1-8 years	Child-report: AUQUEI; Parent-report: QUALIN	A monthly injection of LAR octreotide was efficient in maintaining glycaemia unchanged, without altering the normal weight-and-growth.The HrQoL evaluations of the children and parents were not able to detect any change after the switch of the new treatment (AUQUEI M=8.0, SD=1.33 at six months vs. M=7.9, SD=1.45 at baseline).
Männistö et al. (12); Finnland	To examine whether the HRQoL is worsened in patients with persistent or transient CHI.	cross-sectional survey	Parent-report: n = 65; child-report: n = 19; age: 3-17 years	KINDL, child-report, parent-report	In self-reports of subjects aged 11–17 years and in parent reports of children aged 3–17 years, Persitant-CHI or Transient-CHI children did not have statistically lower scores in any of the six dimensions (physical well-being, emotional well-being, self-esteem, family, friends, and school) or in total scores compared to the reference values.
Yamada et al. (14); Japan	To investigate the incidence, treatment details and outcomes of patients with endogenous hyperinsulinemic hypoglycemia (EHH), including those with transient/persistent CHI.	Retrospective survey	n = 447 patients with CHI	Physician-reported retrospective HrQoL	Findings indicated an improvement in the prognosis of persistent CHI over the past 10 years. However, frequent post-treatment residual hypoglycemia and impaired quality of life were reported too.
Shah et al. (13); United Kingdom	To report the first case on the use of lanreotide in an adolescent girl with diazoxide-responsive CHI.	case report, pre post design	n = 1; age: 14 yreas	PedsQL, child-report, parent-report	On Diazoxide, both parents and child reported a clinically significantly lower HrQoL with respect to psychosocial aspects in the PedsQL There was a significant improvement in HrQoL after 1 year on Long-Acting Somatostatin Analogue

AUQUEI, Pictured Child’s Quality of Life Self Questionnaire; KINDL-R, Revised questionnaire to assess Health-Related Quality of Life in children and adolescents; PedsQL, Pediatric Quality of Life Inventory; CHI, Congenital hyperinsulinism; n, number; QUALIN, Infant Quality of Life; HrQoL, Health-related Quality of Life; p, significance.

### HrQoL in Children and Adolescents Diagnosed With CHI

In total, two studies were of an observational nature, i.e. aimed at describing HrQoL of children and adolescents with CHI. Männistö and colleagues investigated the impact of transient (n=32) and persistent (n=33) CHI on the HrQoL of 3–17-year-old patients recruited from the Finnish CHI registry (12). Transient CHI was defined as a neonatal onset of hyperinsulinism followed by successful discontinuation of medication within four months. Parents were asked to rate their children’s HrQoL using the revised KINDL-R. Children and adolescents aged 11 years and older were also given a self-report version of the questionnaire. The results showed that there were no notable differences between the two groups. However, differences were found when compared to the age- and gender-specific reference values of the instrument. In the persistent CHI group, the scores were significantly higher in self-reports in the dimensions physical well-being (p < 0.001), self-esteem (p=0.002), and total scores (p=0.038). In contrast, self-esteem (p=0.021) was rated higher in parent reports. The scores of the transient CHI group were statistically higher in self-reported school-related well-being (p=0.032). In comparison, scores were higher in physical well-being (p<0.001) and total scores (p=0.013) in parent reports. The authors note that the results reflect improved management of recently born and treated CHI patients, as none of the included patients underwent subtotal pancreatectomy, which is associated with a high risk of insulin-dependent diabetes and pancreatic exocrine dysfunction, and none of the patients had an intellectual disability due to a severe hypoglycaemic insult. Furthermore, results might reflect the high number of patients who were able to discontinue treatment.

Yamada et al. chose a different data collection strategy (14). In a nationwide survey of clinics, those who had treated patients with endogenous hyperinsulinemic hypoglycaemia between 2017 and 2018 were asked to characterize these patients in more detail. In total, 447 patients with CHI were identified in this survey [transient CHI (n = 197), persistent CHI (n = 225), and unknown subtype (n = 25)]. The questionnaire concerning CHI patients included questions on socio-demographic information, treatment details, and post-treatment outcomes, including HrQoL. However, it was not explained how the external assessment of HrQoL was obtained. A detailed description of the HrQoL of CHI patients was not provided to the readers, only the statement that HrQoL was frequently impaired post-treatment in patients with endogenous hyperinsulinemic hypoglycaemia.

Two studies had a more experimental approach. Le Quan Sang et al. reported outcomes for ten children aged one to eight years, in whom the three daily subcutaneous octreotide injections were replaced by a single and monthly intramuscular injection of long-acting release (LAR) octreotide (11). The participants’ HrQoL was assessed using the AUQUEI picture questionnaire for children aged three to eight years and the QUALIN parental report questionnaire. HrQoL was measured at the first injection visit and six months later. No change in either child-reported or parent-reported HrQoL was found in this period. However, satisfaction with the new treatment option was rated high by all parents.

In their case report, Shah and colleagues described how HrQoL of a 14-year-old patient improved significantly after one year of treatment with subcutaneous injections of long-acting somatostatin analogue compared to the previous treatment with orally administered diazoxide (13). In addition to the PROM PedsQL reported by the parents and child, a semi-structured interview was conducted with the family. The patient reported that she was bullied during diazoxide treatment because of the excessive hair growth it caused and that her adherence to the treatment was poor as a result of that. With the switch to monthly injection of LAR octreotide, these side effects disappeared and the patient became more satisfied overall. Both the parents and the patient reported a significant improvement in HrQoL after one year on the new treatment.

## Discussion

This scoping review identified four articles describing HrQoL in children and adolescents diagnosed with CHI. Only one study compared HrQoL scores with reference values. Two other studies addressed the change in HrQoL resulting from new treatment procedures. Furthermore, the fourth study described the retrospective physician reported post-treatment HrQoL of CHI patients treated for hypoglycaemia in hospitals. The results described are inconclusive; however, they are difficult to compare due to their different study designs and the different methods of measuring HrQoL.

The compilation of studies illustrates that research on HrQoL in patients with CHI is still in its early stages. More observational studies with larger sample sizes and longitudinal designs are needed to profoundly understand whether and how CHI affects HrQoL in children and adolescents. In this context, more detailed insights, e.g., the extent to which disease activity and adaption to the condition impacts HrQoL in patients with CHI, should also be investigated in more detail. Validated PROMs should be used for this purpose. The identified HrQoL studies used validated generic HrQoL instruments, specifically, the KINDL-R, PedsQL, QUALIN, and AUQUEI questionnaires in three out of four studies. The advantage of these instruments is that comparisons with the general population or populations affected by other health conditions are possible (16).

Regarding clinical research, it should be noted that HrQoL played a rather minor role in the clinical trials we identified in our search. In one study, HrQoL was included as a secondary outcome in a clinical trial with a pre-post study design (11). As noted in our literature search, clinical outcomes, such as glycaemias or neurodevelopmental outcomes, have been studied more frequently. However, there is no question that PROMs are also essential in clinical research. In particular, where evidence of economic benefit is critical to the success of orphan drug reimbursement, robust PROM data are crucial (17).

PROMs were initially used at an aggregate level, as in the observational and clinical studies identified in this review. However, PROMs are also becoming increasingly important in clinical practice. By asking routine screening questions, physicians can use the results of PROMs as a basis for the consultation, which can help improve communication between physicians and patients (18). Moreover, PROMs can be collected on an ongoing basis to monitor patient progress and identify problems such as disease progression and possible side effects of prescribed treatments. Studies have shown that the routine use of PROMs not only increases patient satisfaction but can also improve patient outcomes, including symptom control and HrQoL (19, 20).

When considering the use of HrQoL in clinical trials or in clinical practice, it is important to be aware of the different attributes of PROMs. Especially for rare diseases, condition specific PROMs can be helpful. Compared to generic PROMs, they address the most specific concerns of patient populations and are more sensitive to condition-specific changes (16). To date, there is no CHI-specific instrument to measure HrQoL. Given the development of novel medical therapies such as LAR octreotide injections and also surgical advances such as laparoscopic pancreatectomy (21), the development of a CHI-specific HrQoL instrument would be an asset to the evaluation of these novel interventions.

In order to develop a CHI-specific HrQoL instrument, qualitative research is needed. By giving patients a voice, it becomes possible to understand the patients experience in depth and to capture the full range of manifestations and their relationship to HrQoL. In one case report included in this review, a semi structured interview was conducted with the adolescent patient and her parents (13). Valuable insights emerged from the interview, such as how the excessive hair growth caused by diazoxide treatment was the reason she was bullied by other children and led to poor treatment adherence. It is testimonials like these, after all, excessive hair growth is a common side effect of diazoxide (21), that are essential for characterizing the disease-specific HrQoL. However, this should be done in a systematic way with clearly defined research questions.

However, there are several issues to consider when developing a CHI-specific HrQoL instrument. The rarity of the condition is a challenge for the development and validation of a PROM. Cost-intensive international studies can increase the sample size needed for validation, but linguistic and cultural differences must be considered (22). At the same time, the heterogeneity of CHI manifestation should be taken into account. Stratification according to age groups could be a helpful strategy. Due to the young age of the patients, parents’ perspective should also be included (23), as was the case in the reviewed studies. For the development of a PROM, which should be preceded by a thorough systematic literature review, the identification of existing PROMs for patients with similar symptoms, such as type 1 diabetes-specific PROMs for CHI patients who have developed insulin-dependent diabetes, could serve as a starting point.

It should be noted that this is a scoping review that does not claim to identify all existing studies on HrQoL in children and adolescents with CHI. It allows us to summarize research findings, draw conclusions from the existing literature about the general state of research activity, and identify gaps in the evidence base where research has not yet been conducted. However, it is essential to bear in mind that quality assessment does not form part of a scoping study (10). Therefore, it is not possible to identify gaps in the literature that are due to poor research quality.

In summary, few studies to date have addressed HrQoL in children and adolescents with CHI. The few studies that do exist yield inconsistent results while different study designs hamper comparisons. More observational studies characterizing HrQoL with mixed-methods approaches are needed. The use of validated generic and condition-specific HrQoL instruments can not only contribute to a deeper understanding of HrQoL of patients but also strengthen clinical research.

## Data Availability Statement

The original contributions presented in the study are included in the article/supplementary material. Further inquiries can be directed to the corresponding author.

## Author Contributions

KK, SW, and JQ developed the study concept and the design. KK and SW screened the publications and identified relevant articles. KK wrote the first draft of the manuscript. SW and JQ revised the first draft critically for important intellectual content. All authors have revised the subsequent drafts critically and agreed to be accountable for all aspects of the work. All authors contributed to the article and approved the submitted version.

## Conflict of Interest

The authors declare that the research was conducted in the absence of any commercial or financial relationships that could be construed as a potential conflict of interest.

## Publisher’s Note

All claims expressed in this article are solely those of the authors and do not necessarily represent those of their affiliated organizations, or those of the publisher, the editors and the reviewers. Any product that may be evaluated in this article, or claim that may be made by its manufacturer, is not guaranteed or endorsed by the publisher.
